# The association between frailty and mortality among lower limb arthroplasty patients: a systematic review and meta-analysis

**DOI:** 10.1186/s12877-022-03369-w

**Published:** 2022-08-24

**Authors:** Yunfeng Bai, Xiao-Ming Zhang, Xiangyu Sun, Jiaming Li, Jing Cao, Xinjuan Wu

**Affiliations:** Department of Nursing, Chinese Academy of Medical Sciences - Peking Union Medical College, Peking Union Medical College Hospital (Dongdan campus), Beijing, 100730 China

**Keywords:** Frailty, Mortality, Total knee arthroplasty, Total hip arthroplasty, Meta-analysis

## Abstract

**Background:**

Some studies associate frailty and postoperative mortality in hip or knee replacement patients, and others have explored the relationship between the frailty index and changes in postoperative mortality in hip or knee replacement patients, but their findings are not consistent. This meta-analysis and systematic review aimed to pool the results of existing studies to explore whether frailty is an independent risk factor for postoperative mortality in patients with lower limb arthroplasty (including hip or knee arthroplasty).

**Methods:**

On December 15, 2021, we searched the relevant articles from the PubMed, Embase, Medline (via Ovid), China National Knowledge Infrastructure (CNKI) and Wan Fang Med Online databases. We used the Newcastle–Ottawa Scale (NOS) to assess the quality of the articles that met the exclusion and inclusion criteria. R Studio was used to analyze the effect sizes (based on the random model integration) on the extracted data. Meanwhile, potential publication bias and sensibility analysis were performed.

**Results:**

We included seven studies, which included a total of 460,594 patients, for quantitative analysis. Overall, frailty increased the risk of mortality in lower limb arthroplasty patients compared to those without frailty, as measured by a pooled risk ratio (RR) of 2.46 (95% confidence interval [CI]: 1.81–3.33). Additionally, subgroup analysis based on population revealed that the pooled RRs for total knee arthroplasty (TKA) patients in three studies and total hip arthroplasty (THA) patients in four studies were 2.61 (95% CI: 2.26–3.02) and 3.18 (95% CI: 1.92–5.28), respectively, for TKA patients in three studies and THA patients in four studies. Additionally, these statistically significant positive associations persisted in subgroup analyses by study design, geographic region, and follow-up period.

**Conclusion:**

Frailty is an independent risk factor for postoperative mortality in patients undergoing lower limb arthroplasty, according to our findings. This suggests that frailty may be a predictor of preoperative risk stratification for patients with such elective surgery and could alert doctors and nurses of early screening and medical care interventions in patients with such a need for surgery to reduce postoperative mortality in lower limb arthroplasty patients.

**Supplementary Information:**

The online version contains supplementary material available at 10.1186/s12877-022-03369-w.

## Background

Lower limb arthroplasty, including hip or knee replacement, is the most common elective inpatient noncardiac surgery in countries with higher economic levels, increasing the number of annual lower limb arthroplasties in elderly patients in the West since 2000 [1]. Primary total hip arthroplasty (THA) is expected to exceed 572,000 procedures in the United States by 2030, and primary total knee arthroplasty (TKA) is expected to exceed 3,480,000 procedures per year [2]. In China, the number of artificial joint replacement operations in 1995 was 20,000 to 30,000 [3], increased to 680,000 in 2018, and increased at a rate of 20% per year [4]. Approximately 60% of orthopedic surgery patients in China undergo THA annually, and the number of operations has increased year by year [5]. While lower limb arthroplasty is typically safe [6, 7], cost-effective, and improves patient quality of life [8, 9], it does carry a risk of significant adverse events (AEs), including pulmonary embolism and death [9, 10]. Increased age on its own is a significant predictor of poor postoperative outcomes after lower limb arthroplasty [10, 11]. However, age does not adequately account for the changes in outcome experienced by surgical patients [12]. One factor that could explain this change is frailty. However, the results of lower limb arthroplasty in physiologically frail individuals are unclear.

Frailty is a broad manifestation of susceptibility to adverse outcomes due to the accumulation of age- and disease-related problems across multiple domains [13, 14]. Frailty increases the risk of mortality and adverse health outcomes [13, 15, 16] due to a person’s vulnerability to stressors [14]. Frail individuals, regardless of their actual age, are more susceptible to stressors such as surgery, increasing their risk of adverse outcomes. Numerous studies have examined the association between frailty and postoperative mortality in patients with lower limb arthroplasty, with the majority demonstrating a clear link between increasing frailty and poor outcomes [17–21]. There was no correlation between frailty and mortality in individuals with lower limb arthroplasty in two trials [22, 23]. Numerous studies have attempted to establish a link between the frailty index and mortality [24–28]. We believe that it is critical to synthesize the evidence on this critical topic. The purpose of this study was to pool data from previous studies to determine whether frailty is an independent risk factor for postoperative mortality in patients undergoing lower limb arthroplasty (including hip or knee arthroplasty) to provide clinicians and nurses with evidence-based recommendations.

## Methods

This meta-analysis adhered to the Preferred Reporting Items for Systematic Reviews and Meta-Analyses Statement guidelines (PRISMA). Our protocol has been registered with PROSPERO (CRD42022303102).

### Search strategy

Five databases were searched independently by two authors (YFB, XYS) from database conception to 14:00 on 15 December 2021: PubMed, Embase, Medline (through Ovid), China National Knowledge Infrastructure (CNKI), and Wan Fang Med Online. Additionally, we combined keywords with medical subject headers (MeSH). The search strategy was as follows: ((Knee Arthroplasty) OR (Total Knee Arthroplasty) OR (“Arthroplasty, Replacement, Knee”[Mesh]) OR (Total Knee replacement) OR (Hip Arthroplasty) OR (Total Hip Arthroplasty) OR (Total Hip replacement) OR (“Arthroplasty, Replacement, Hip”[Mesh])) AND ((frail*) OR (“frailty” [Mesh])). Additionally, we attempted to locate relevant studies through references and conducted a Google search for gray literature. Supplementary file 1 contains a detailed search strategy for PubMed.

### Inclusion and exclusion criteria

We included all observational studies examining the links between frailty and mortality in patients undergoing lower limb arthroplasty. We eliminated commentaries, reviews, conference proceedings, correspondence, editorials, letters to the editor, and case reports. Additionally, we retained five papers that detailed the outcomes of studies that used frailty scores as continuous variables.

### Study selection process

Following the storage of all relevant papers in the appropriate format, two writers screened articles using Endnote software (Clarivate Analytics, USA). First, duplicate articles were deleted using Endnote software’s functionality and then swiftly perused the article’s title and abstract section, eventually discovering entire texts that satisfied the inclusion and exclusion criteria. When a contentious piece was met, the third author was included in the decision-making process.

### Data extraction

The two authors (YFB and XMZ) separately retrieved the papers’ basic information and several characteristics associated with frailty and mortality (such as tools to assess frailty, effect size, etc.). When two authors disagreed about the extracted content, the third author rendered the decision. Table 1 contains the retrieved data in detail.

**Table 1:** Summary of Included Studies on frailty associated with mortality among lower limb arthroplasty patients

Author	Design	County	Male%	Setting	Prevalence of frailty	Sample size	Age/years	Frailty Criteria	Effect measures	Outcome assessed
Laursen 2021 [23]	RCS	Denmark	56%	Hospital	20%	284	74(67–80)	modified Frailty index	RR	90-day mortality
Johnson 2021 [17]	RCS	USA	43.5%	Hospital	23%	5341	Aged> 50 years	Frailty index	HR	1-year mortality
Ferguson 2021 [22]	PCS	UK	38.8%	Hospital	37.4%	6682	Aged> 65 years	electronic Frailty index	RR	90-day mortality
Schwartz 2021 [21]	RCS	USA	38%	Hospital	19.8%	179,702	66.4 ± 6.6	modified Frailty index	RR	30-day mortality
R.L Johnson 2018 [20]	PCS	USA	48.8%	Hospital	22.7%	8640	Aged> 50 years	Frailty index	HR	90-day mortality
Mclsaac D. I 2016 [19]	RCS	Canada	39%	Hospital	2.4%	125,163	74(6)	ACG	HR	1-year mortality
Mclsaac 2016 [18]	RCS	Canada	44.4%	Hospital	3.1%	134,782	Aged> 65 years	ACG	HR	1-year mortality
R.L Johnson 2021 [24]	PCS	USA	45.8%	Hospital	NA	18,397	68(61,75)	Frailty index	HR	In-hospital mortality
Runner 2017 [25]	RCS	USA	37.2%	Hospital	NA	90,260	70.75 ± 7.17	modified Frailty index	OR	In-hospital mortality
Shin 2016 [26]	RCS	USA	40.5%	Hospital	NA	39,807	Aged> 18 years	modified Frailty index	OR	In-hospital mortality
Sophia 2018 [27]	RCS	USA	55.2%	Hospital	NA	140,158	64.8	modified Frailty index	OR	In-hospital mortality
Traven 2019 [28]	RCS	USA	41.1%	Hospital	NA	16,304	65.2	modified Frailty index	OR	In-hospital mortality

*RCS* Retrospective cohort study, *PCS* prospective cohort study, *ACG* Johns Hopkins Adjusted Clinical Groups frailty-defining diagnoses indicator, *HR* hazard ratio, *OR* odd ratio, *RR* risk ratio

### Quality assessment

Two authors (YFB and XMZ) assessed quality bias using the Newcastle–Ottawa Scale (NOS), a commonly used tool for cohort studies. This scale assigns a numerical value to the study’s quality based on how many stars it receives. The overall assessment scale varied from 0 to 9, with more stars indicating a higher-quality study. Quality is classified as poor (0–4), moderate (5–7), or high (> 7).

### Statistical analysis

Two independent writers (YFB and JML) retrieved and assessed the effect sizes (hazard ratios and risk ratios) associated with frailty and mortality using Microsoft Excel. The heterogeneity between studies was denoted by *I*^*2*^ and discovered using Cochran’s Q test. The threshold for significance in the category of heterogeneity was defined as *I*^*2*^ > 50% and *I*^*2*^ < 50%. Due to the variability of the study design and the frailty assessment scale, we calculated effect sizes using both fixed and random effect models. Then, the most appropriate model was selected for calculating and summarizing the relative risk of frailty and mortality with a 95% confidence interval. Additionally, subgroup analyses were conducted according to demographics, geographic region, study design, follow-up time, and frailty evaluation scales. Cone plots were used to undertake publication bias and sensitivity assessments.

### Search results and study characteristics

**Fig. 1:**
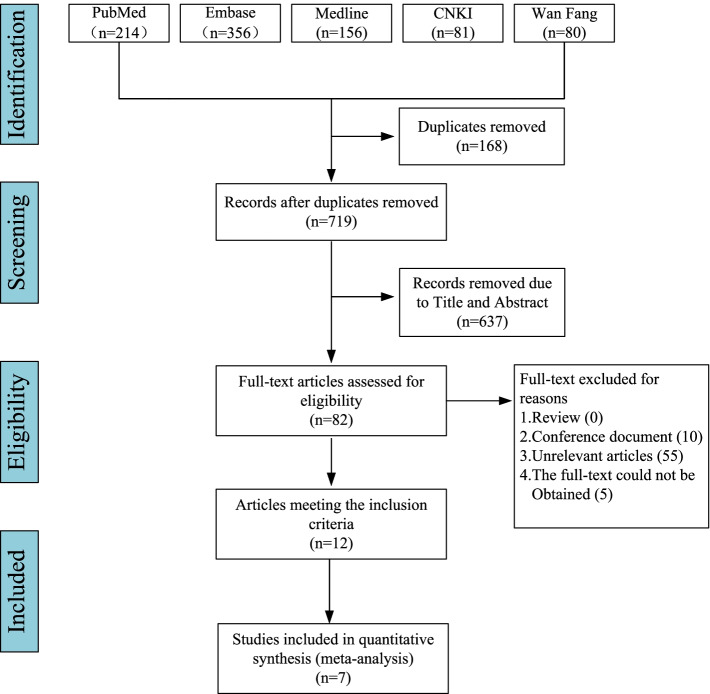
Research screening flowchart

We found 887 relevant articles from the PubMed (214), Embase (356), Medline (156), CNKI (81) and Wan Fang (80) databases. After deleting duplicates with Endnote software, 719 articles remained. Two writers reviewed the titles and abstracts of the publications included in the study to determine which were most closely linked to the study. There are still 82 articles left to qualify for a more detailed assessment. Therefore, after reading the full text, seven studies were included in the quantitative analysis, and five studies were included in the description analysis. Each of the twelve studies discussed above met the predefined inclusion and exclusion criteria. Figure 1 illustrates the specific reasons for exclusion.

Five studies involving 304,926 participants examined the association between frailty and mortality. Seven investigations measured the association between frailty and mortality, involving a total of 460,594 participants (shown in Table 1). Overall, through the investigator including patients in the full-age group, the average patient age in most studies remained older than 65 years. Three studies [20, 22, 24] were prospective cohort studies, whereas the remaining six were retrospective cohort studies [17–19, 21–23, 25–28]. The countries represented ranged from the United States to Europe, with eight in the United States [17, 20, 21, 24–28], one in the United Kingdom [22], one in Denmark [23], and two in Canada [18, 19]. All these studies were conducted in hospital settings [17–28]. The prevalence of frailty ranged from 3.1 to 37.4%. Most studies reported on in-hospital mortality, with one reporting on 30-day mortality, three reporting on 90-day mortality, and three reporting on 1-year mortality. Canada [18] had the largest sample size of 202,980 patients, while Denmark [23] had the smallest sample size of 284 individuals. Six studies [21, 23, 25–28] utilized the modified frailty index (mFI) to assess frailty, one used the electronic frailty index (eFI) [22], three used the frailty index [17, 20, 24], and two used the Johns Hopkins adjusted clinical groups frailty-defining diagnostic indicator (ACG) [18, 19].

### Meta-analysis of the mortality effects of frailty

To make the results more reliable, we followed the study of the relevant researchers in the statistical field [29, 30], unifying the risk ratio (RR) values and the hazard ratio (HR) values, both converted to risk ratio (RR) values.

Because the risk ratio (RR) values do not obey a normal distribution, to make the data more stable and weaken the model heteroscedasticity and collinearity, the RR values and confidence intervals are taken as a natural logarithm before merging the calculation.

**Fig. 2:**
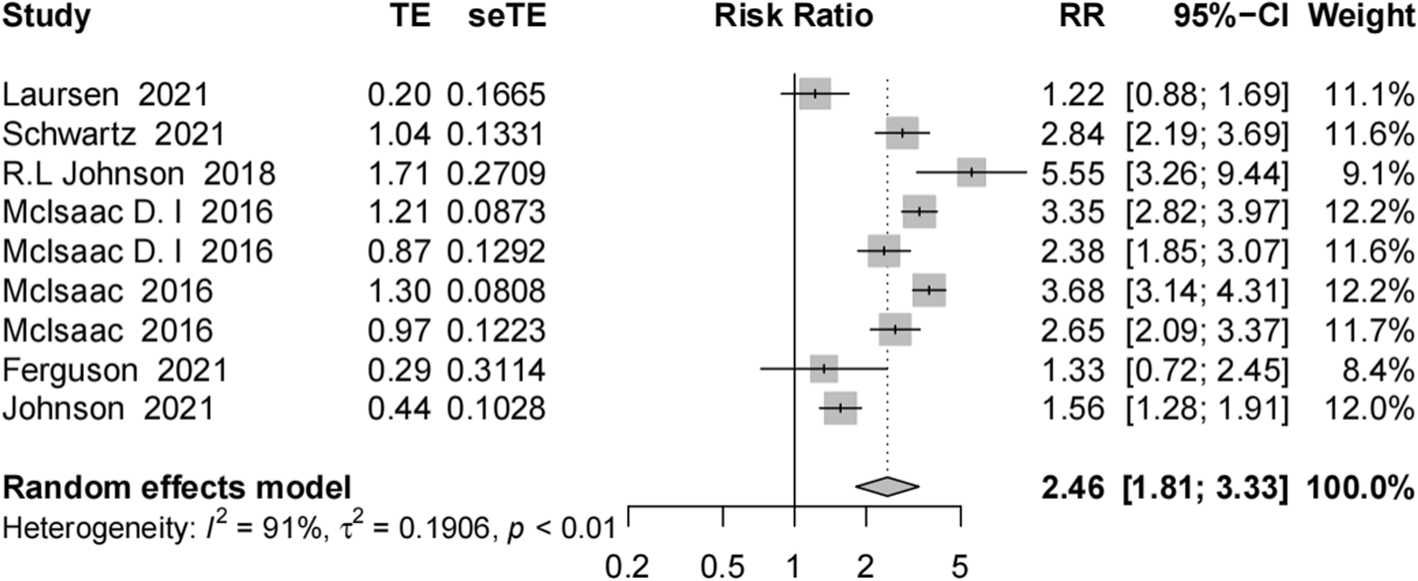
Meta-analysis of the effects of frailty on mortality among lower limb arthroplasty patients

The meta-analysis included seven studies, of which two focused exclusively on operation methods. For the calculations, we consider these to be distinct studies. The pooled RR value was 2.46 (95% confidence interval [CI]: 1.81–3.33) for frail patients versus those with lower limb arthroplasty who were not frail, indicating that frailty can be an independent predictor of mortality in patients with lower limb arthroplasty (Fig. 2).

### Subgroup analysis was conducted using data from various populations

**Fig. 3:**
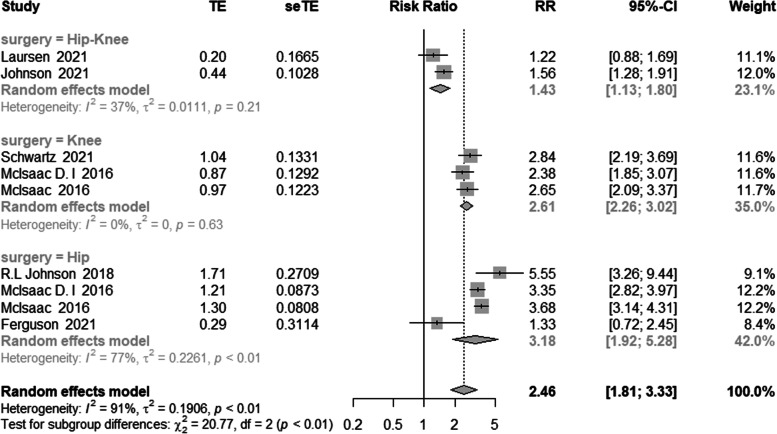
Meta-analysis of the effects of frailty on mortality based on different population

Two studies examined the association between frailty and mortality in patients undergoing lower limb arthroplasty (including THA and TKA) (pooled RR = 1.43, 95% confidence interval [CI]: 1.13–1.80). In isolation, TKA patients who were frail had a 2.61-fold increased risk of mortality compared to no frail patients (pooled RR = 2.61, 95% CI: 2.26–3.02; three studies). Meanwhile, THA patients who were frail had a 3.18-fold increased risk of mortality compared to patients who were not frail (assessed in four studies, pooled RR = 3.18, 95% confidence interval [CI]: 1.92–5.28) (Fig. 3).

### Subgroup analysis was conducted using a variety of frailty assessment scales

**Fig. 4:**
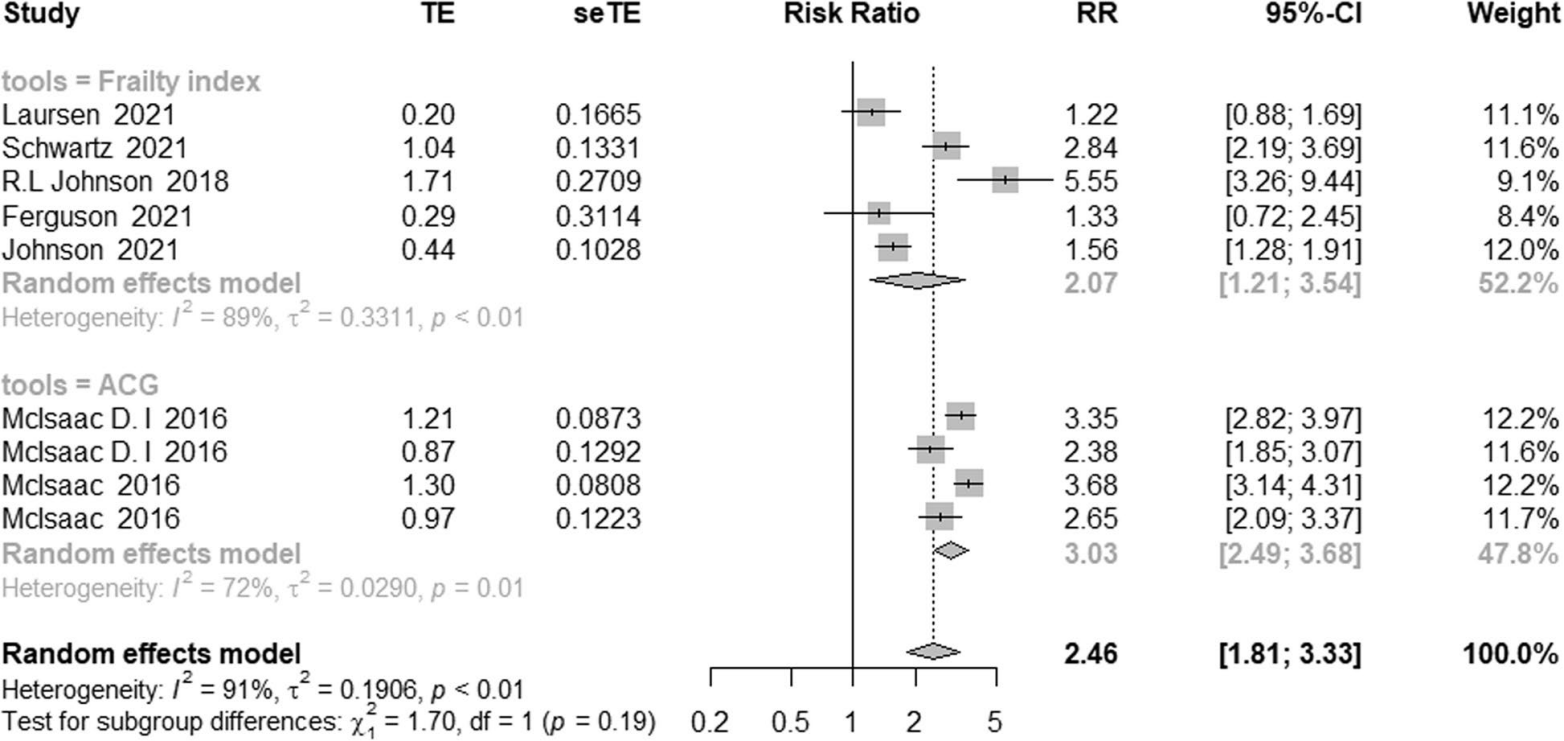
Meta-analysis of the effects of frailty on mortality based on different assessment tools

By comparing the frailty index (FI) to another tool, we were able to perform a subgroup analysis of the frailty assessment tool. Because different researchers chose different frailty indexes, such as the modified frailty index and electronic frailty index, we merged different types of frailty indexes into a group. When five studies using FI were pooled, the pooled relative risk of frailty among patients who died was 2.07 (95% confidence interval [CI]: 1.21–3.54). Another four studies that used ACG to assess frailty had a pooled RR of 3.03 (95% CI: 2.49–3.68), as shown in Fig. 4.

### Analysis of subgroups according to study design, geographic region, and type of follow-up

**Fig. 5:**
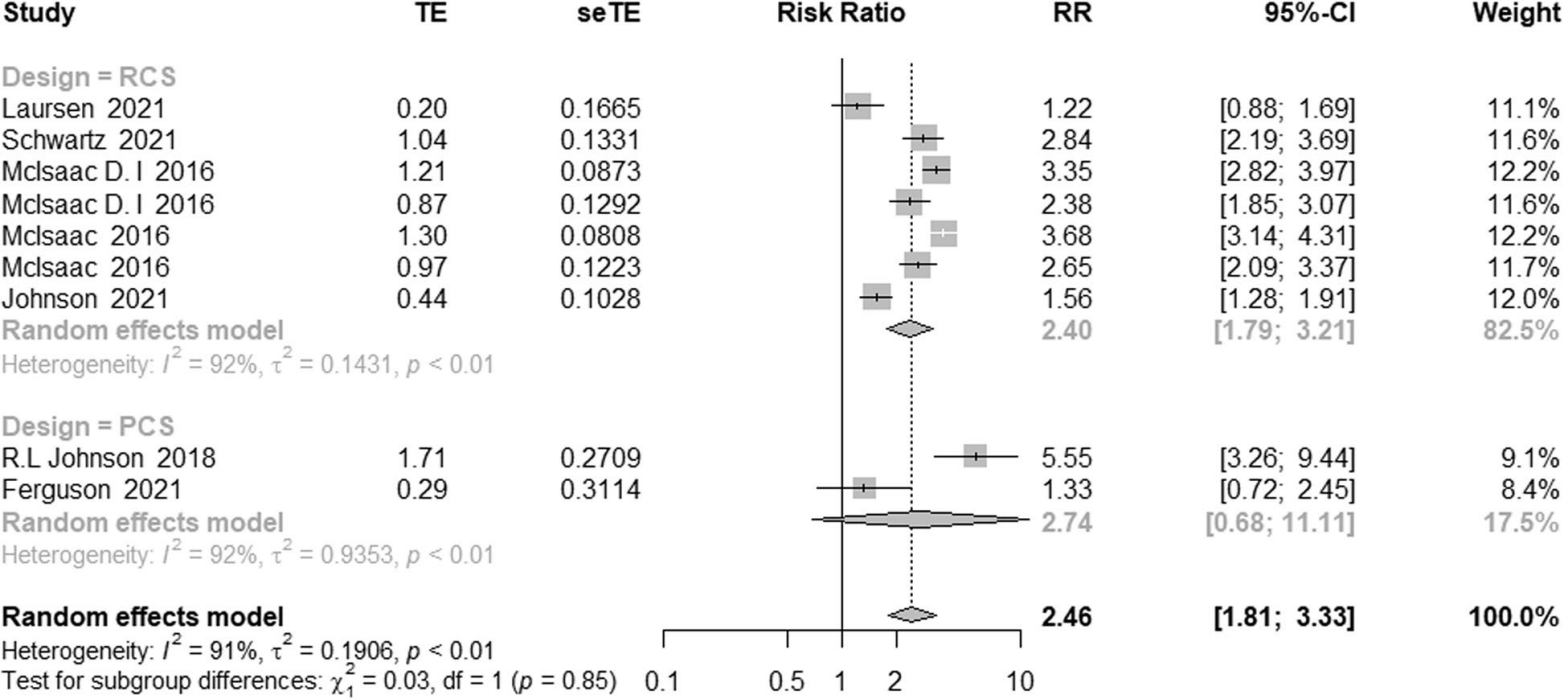
Meta-analysis of the effects of frailty on mortality based on different study design

Seven of the studies were RCS, and the remaining studies were PCS; hence, we performed a subgroup analysis by research type. Across cohort studies, the findings demonstrated a statistically significant link between frailty and mortality (RR = 2.40, 95% CI: 1.79–3.21). Similar findings were observed in the prospective cohort study (RR = 2.74, 95% confidence interval [CI]: 0.68–11.11), as illustrated in Fig. 5.

**Fig. 6:**
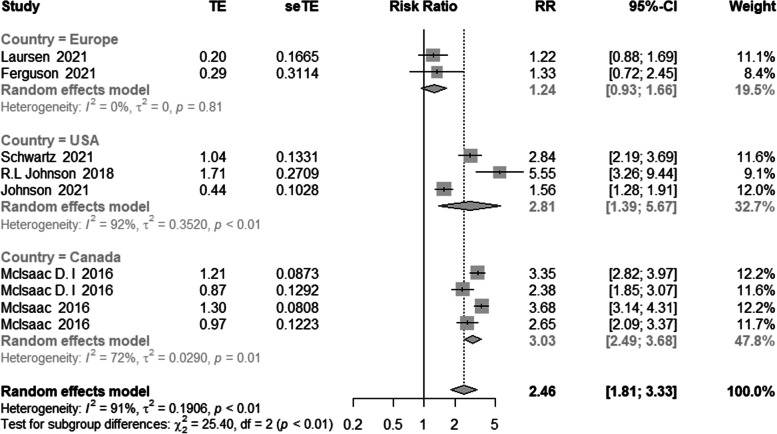
Meta-analysis of the effects of frailty on mortality based on different geographic region

Additionally, we conducted a subgroup analysis by geographical region. The results indicated that the associations were stronger in the United States of America (3 studies) and Canada (4 studies) than in Europe (2 studies). Patients who are frail suffer progressively excess mortality compared to patients who are not frail following lower limb arthroplasty (shown in Fig. 6).

**Fig. 7:**
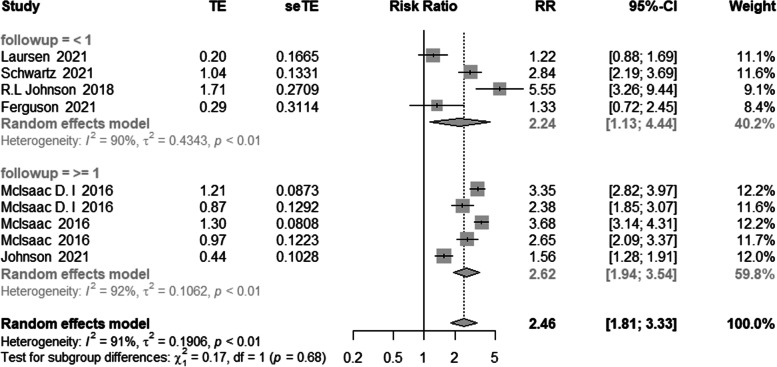
Meta-analysis of the effects of frailty on mortality based on follow-up

Additionally, subgroup analysis using follow-up data revealed that there was no change in the link between frailty and mortality (shown in Fig. 7).

### Quality assessment

According to the Newcastle–Ottawa Scale’s criteria, the majority of research scored seven stars, while three scored eight (Table 2).

**Table 2:** Results of quality assessment using the Newcastle-Ottawa scale quality for cohort studies

Newcastle-Ottawa scale	Selection (1)				Comparability (2)	Outcome (3)			Total (9)
	Representativeness of the exposed cohort	Selection of the non-exposed cohort	Ascertainment of exposure	Demonstration that outcome of interest was not present at start of study	Comparability of cohorts on the basis of the design or analysis	Assessment of outcome	Was follow-up long enough for outcome to occur?	Adequacy of follow-up of cohorts	
Laursen 2021 [23]	☆	☆	☆	☆	☆☆	☆	–	☆	8
Johnson 2021 [17]	☆	☆	☆	☆	☆	☆	–	☆	7
Ferguson 2021 [22]	☆	☆	☆	☆	☆	☆	–	☆	7
Schwartz 2021 [21]	☆	☆	☆	☆	☆	☆	–	☆	7
R.L Johnson 2018 [20]	☆	☆	☆	☆	☆	☆	–	☆	7
Mclsaac D. I 2016 [19]	☆	☆	☆	☆	☆	☆	–	☆	7
Mclsaac 2016 [18]	☆	☆	☆	☆	☆	☆	–	☆	7
R.L Johnson 2021 [24]	☆	☆	☆	☆	☆	☆	–	☆	7
Runner 2017 [25]	☆	☆	☆	☆	☆	☆	–	☆	7
Shin 2016 [26]	☆	☆	☆	☆	☆☆	☆	–	☆	8
Sophia 2018 [27]	☆	☆	☆	☆	☆☆	☆	–	☆	8
Traven 2019 [28]	☆	☆	☆	☆	☆	☆	–	☆	7

### Analyses of sensitivity and probable publication bias

Begg’s test was used to examine whether there was any potential bias in publishing, and the results indicated that there was none (*p* = 0.2971). (Shown in Supplemental Fig. 1 and Fig. 2). Additionally, we performed a leave-one-out sensitivity analysis, which demonstrated that our study was steady and robust (shown in Supplement Fig. 3).

## Discussion

We discovered that lower limb arthroplasty patients who were frail had a greater risk of mortality than those who were not frail and that this risk increased in lockstep with the rising frailty index. Additionally, this fatality risk is unrelated to the study’s design, geographic location, or other variables. This finding implies that frailty may be a risk factor (or a predictive factor) for postoperative mortality in individuals undergoing lower limb arthroplasty. It can provide theoretical support for clinicians to predict postoperative mortality in lower limb arthroplasty patients and to use a frailty index to assess stratified high-risk inpatients and to remind physicians and nurses to pay more attention to such patients. This is the first meta-analysis that we are aware of that examines the association between disability and mortality in lower limb arthroplasty patients. Given the aging of the population, the number of future patients undergoing lower limb arthroplasty surgery is likely to increase year after year, and frailty screening can assist clinicians in developing a complete predictive tool for forecasting mortality and commencing early intervention to ameliorate frailty syndrome in terms of reducing mortality in lower limb arthroplasty patients. Lower limb arthroplasty is an elective procedure that is only performed on medically healthy patients who are surgically fit. The association between frailty and poor outcomes emphasizes the fact that frailty assesses areas that are not covered by routine preoperative medical examination.

Most of the articles included in this study focused on outcomes that occurred 90 days to 1 year after surgery. While immediate effects are critical, lower limb arthroplasty is intended to be a long-term strategy that improves pain and function in the long term. As a result, it will be critical to discover long-term, patient-centered, clinically meaningful postoperative outcomes associated with frailty, as well as the possibility that preoperative frailty may be connected with long-term postoperative health-related quality of life [31].

Five articles included the frailty index as a continuous variable to explore the increased mortality of patients for each 1-point increase in the frailty index. All the results showed a stepped increase in patient mortality with the frailty index [24–28]. All their studies ended with 30-day mortality, and although we currently see an associated outcome, we still need more studies that extend the observation period to determine whether this outcome is stable.

Although various prior studies have explored the processes behind the correlation between frailty and mortality, this association remains unsolved due to the presence of multiple complex components. Numerous explanations are possible. First, frail patients with more vulnerable conditions demonstrate a variety of visible deficits, such as decreased physical reserve, chronic malnutrition, and cognitive impairment, all of which raise the risk of undesirable consequences when patients are exposed to substantial negative stress, such as surgery. Second, fragile processes that involve chronic inflammatory and proinflammatory cytokines, such as C-reactive protein or interleukin-6, increased the chance of patients dying postoperatively. Additional study is necessary to gain a better understanding of the frailty process and to assist physicians in initiating early intervention.

When the frailty index and other frailty evaluation measures were applied, our subgroup analysis demonstrated that debilitating can be an independent predictor of mortality risk. Numerous vulnerability assessment tools have been implemented in a variety of contexts, each with its own set of benefits and drawbacks. The best vulnerability score screening procedure should be simple, sensitive, and accessible. We discover that the frailty index (35 items) and its several versions, such as the modified frailty index and electronic frailty index, are quite dependable when used to assess frailty. However, we must keep in mind that some critical frailty evaluation techniques, such as the clinical frailty scale (CFS), have not been used for lower limb arthroplasty patients in relevant studies, which may alter the outcomes of the examination of subgroups utilizing the frailty assessment method. Additionally, other subgroup studies utilizing different designs and countries produced equivalent findings, demonstrating that the relationship between frailty and mortality is constant and persistent in patients with lower limb arthroplasty.

There are certain advantages and disadvantages to our systematic review and meta-analysis. To our knowledge, this is the first meta-analysis study to encompass 460,594 participants and to employ a comprehensive analysis approach to investigate the link between frailty and mortality in lower limb arthroplasty patients. Our work may contribute to resolving the debate over whether frailty can be used to stratify lower limb arthroplasty patients. Our findings imply that, on its own, frailty is a substantial predictor of survival. There are, however, significant limits, and we should proceed with caution when drawing conclusions. First, there are two studies that combine TKA and THA patients, and future trials should focus exclusively on TKA and TKA patients, as these populations differ significantly. Patients undergoing TKA are typically younger, have a higher BMI and are more likely to be female [32], which may explain why fragile therapies have thus far concentrated on THA. Second, there is no gold standard for vulnerability; a 2016 evaluation revealed 79 distinct vulnerability assessment methods, and recent studies frequently suggest new definitions and assessment techniques [33]. By establishing a standard, consistent paradigm for implementing vulnerability, we may not only differentiate vulnerability from related concepts such as comorbidities but also improve cross-validation between diseases and populations.

## Conclusion

This systematic review and meta-analysis summarize the evidence for the effect of frailty on mortality in lower limb arthroplasty patients, indicating that frailty is associated with an increased risk of mortality in lower limb arthroplasty patients compared to no frail lower limb arthroplasty patients and that this association is independent of factors such as geographical region and study design. In addition, mortality in lower limb arthroplasty patients increased with the frailty index. In general, frailty screening may assist clinicians in stratifying risk categories in older lower limb arthroplasty patients, hence assisting clinical healthcare professionals in managing and balancing patient interests and risks. As a result, this group requires diverse and effective medical treatment or interventions to prevent patient mortality.

## Supplementary Information

**Additional file 1: Supplement Fig. 1.** Publication bias was reported using the Begg test.

**Additional file 2: Supplement Fig. 2.** Publication bias was reported using the funnel graph.

**Additional file 3: Supplement Fig. 3.** Graph of sensitivity analysis.

## Data Availability

All data generated or analyzed during this study are included in this published article [and its supplementary information files].

## Abbreviations

CNKI: China National Knowledge Infrastructure
NOS: Newcastle–Ottawa Scale
RR: Risk ratio
CI: Confidence interval
TKA: Total knee arthroplasty;
THA: Total hip arthroplasty
mFI: Modified frailty index
eFI: Electronic frailty index;
FI: Frailty index
ACG: Johns Hopkins adjusted clinical groups frailty-defining diagnostic indicator
HR: Hazard ratio
CFS: Clinical frailty scale
