# Supplementary figures and images for: The association between frailty and mortality among lower limb arthroplasty patients: a systematic review and meta-analysis
Source: BMC Geriatr. 2022 Aug 24;22:702. doi: 10.1186/s12877-022-03369-w (PMC9400276; doi:10.1186/s12877-022-03369-w)

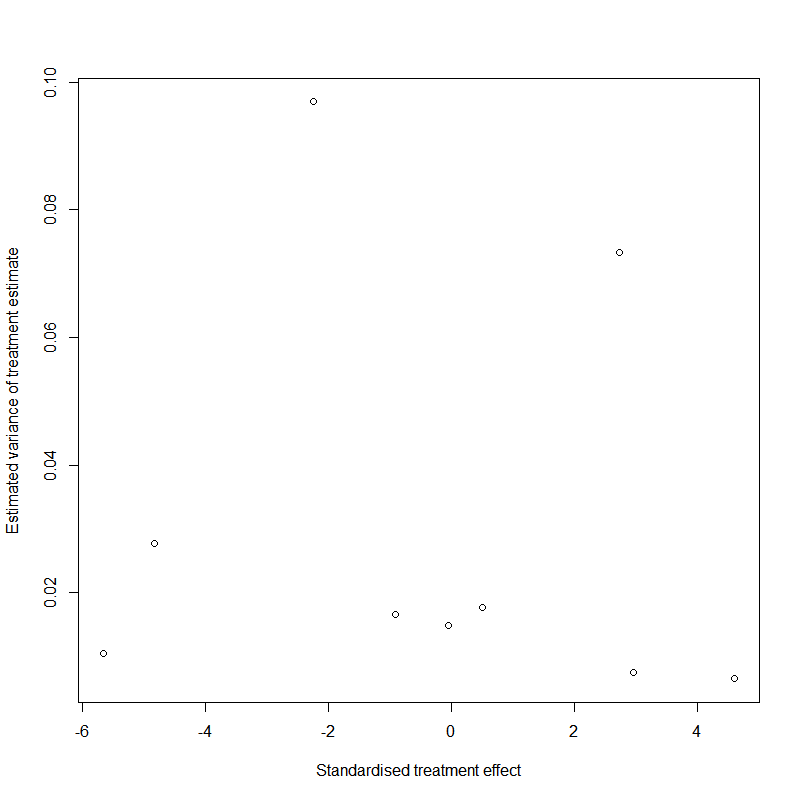

supplement **Fig.1** Publication bias was reported using the Begg test

Supplement: Supplementary file 1 — Additional file 1: Supplement Fig. 1. Publication bias was reported using the Begg test. [file 12877_2022_3369_MOESM1_ESM.docx]

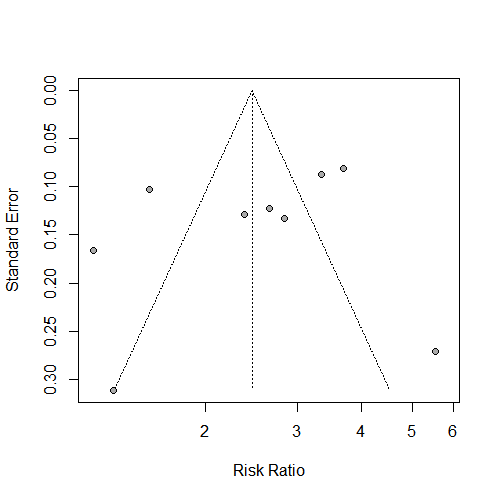


supplement **Fig.2** Publication bias was reported using the funnel graph

Supplement: Supplementary file 2 — Additional file 2: Supplement Fig. 2. Publication bias was reported using the funnel graph. [file 12877_2022_3369_MOESM2_ESM.docx]

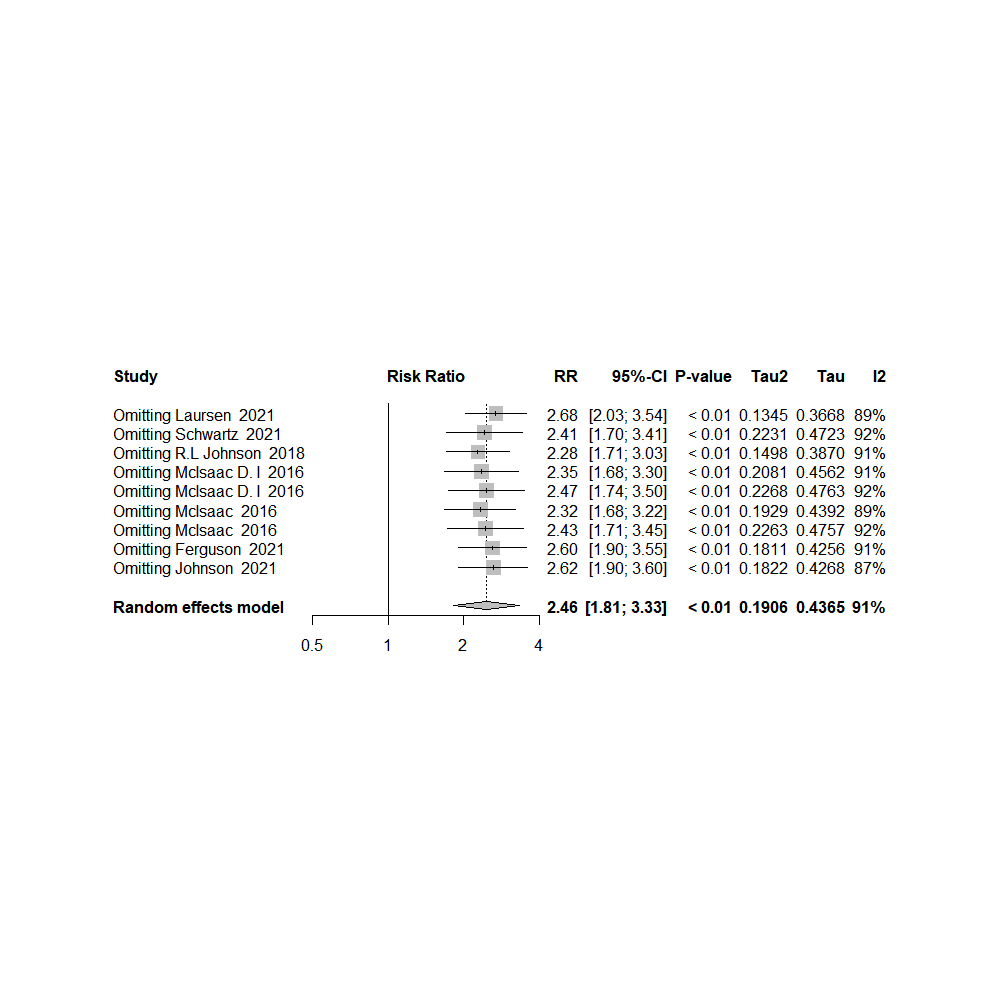


supplement **Fig.3** Graph of sensitivity analysis

Supplement: Supplementary file 3 — Additional file 3: Supplement Fig. 3. Graph of sensitivity analysis. [file 12877_2022_3369_MOESM3_ESM.docx]
